# Supplementary material for: DFT Study on the CO2 Reduction to C2 Chemicals Catalyzed by Fe and Co Clusters Supported on N-Doped Carbon
Source: Nanomaterials (Basel). 2022 Jun 29;12(13):2239. doi: 10.3390/nano12132239 (PMC9268301; doi:10.3390/nano12132239)
Supplement: Supplementary file 1 [file nanomaterials-12-02239-s001.zip › nanomaterials-1748553-supplementary.pdf]

nanomaterials

MDPI

Article

DFT Study on the CO_2 Reduction to C_2 Chemicals Catalyzed by Fe and Co Clusters Supported on N-Doped Carbon

Qian Xue [1], Xueqiang Qi [1,2,*], Tingting Yang [1], Jinxia Jiang [3,*], Qi Zhou [1], Chuang Fu [1] and Na Yang [2,*]

1 School of Chemistry and Chemical Engineering, Chongqing University of Technology, Chongqing 400054, China; xueq@stu.cqut.edu.cn (Q.X.); Tingty@stu.cqut.edu.cn (T.Y.); QiZhou@stu.cqut.edu.cn (Q.Z.); ChuangFu@stu.cqut.edu.cn (C.F.)
2 Chongqing Key Laboratory of Chemical Process for Clean Energy and Resource Utilization, School of Chemistry and Chemical Engineering, Chongqing University, Chongqing 400044, China
3 Chongqing Medical and Pharmaceutical College, Chongqing 400020, China
* Correspondence: xqqi@cqut.edu.cn (X.Q.); jiang106@cqu.edu.cn (J.J.); yna@cqu.edu.cn (N.Y.)

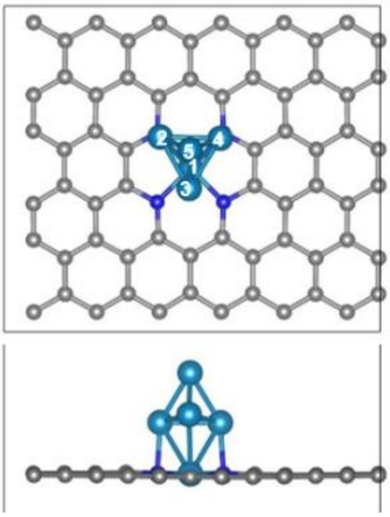

Figure S1. The optimized structures of Co5 supported on four nitrogen doped carbon (NC). Number shown in structures labels the Co atoms.

The Related Reactions in This Paper are Listed as Follows:

The 2-electron direct pathway for the reduction of CO_2 to CO on Fe_n clusters supported by nitrogen-doped carbon was investigated according to the following reaction steps:

$$CO_2(g) + * + \frac{1}{2}H_2 \rightarrow * COOH \quad (S1)$$

$\Delta G_1 = \Delta G(*COOH) - \Delta G(1/2H_2) - \Delta G(*) - \Delta G(CO_2)$

$$* COOH + \frac{1}{2}H_2 \rightarrow * CO + H_2O \quad (S2)$$

$\Delta G_2 = \Delta G(*CO) + \Delta G(H_2O) - \Delta G(1/2H_2) - \Delta G(*COOH)$

$$* CO \rightarrow CO(g) + * \quad (S3)$$

$\Delta G_3 = \Delta G(*) + \Delta G(CO) - \Delta G(*CO)$

Direct Pathway of CO_2RR on Fe_5 and Co_5-nitrogen-doped carbon. A direct 12-electron pathway of CO_2RR on nitrogen-doped carbon-supported Fe_5 and Co_5 cluster has been investigated according to the following reaction steps:

$$2CO_2(g) + * + H_2 \rightarrow * 2COOH \tag{S4}$$

$$* 2COOH + H_2 \rightarrow 2H_2O(l) + * 2CO \tag{S5}$$

$$* 2CO + \frac{1}{2}H_2 \rightarrow * CO - COH \tag{S6}$$

$$* CO - COH + \frac{1}{2}H_2 \rightarrow * C_2O + H_2O(l) \tag{S7}$$

$$* C_2O + \frac{1}{2}H_2 \rightarrow * CHCO \tag{S8}$$

$$* CHCO + \frac{1}{2}H_2 \rightarrow * CHCHO \tag{S9}$$

$$* CHCHO + \frac{1}{2}H_2 \rightarrow * CH_2CHO \tag{S10}$$

Ethylene

$$* CH_2CHO + \frac{1}{2}H_2 \rightarrow C_2H_4(g) + * O \tag{S11}$$

Ethanol

$$* CH_2CHO + \frac{1}{2}H_2 \rightarrow * CH_3CHO \tag{S13}$$

$$* CH_3CHO + \frac{1}{2}H_2 \rightarrow * CH_3CH_2O \tag{S13}$$

$$* CH_3CH_2O + \frac{1}{2}H_2 \rightarrow * CH_3CH_2OH \tag{S14}$$
